# Supplementary material for: Empowering global disease surveillance with CURED: a tool for rapid identification of unique genomic biomarkers
Source: mSystems. 2026 Mar 19;11(4):e01063-25. doi: 10.1128/msystems.01063-25 (PMC13098284; doi:10.1128/msystems.01063-25)
Supplement: Supplemental material — Supplemental methods and figures and captions for Table S1 to S3. [file msystems.01063-25-s0001.docx]

CURED: Supplementary Material

[Installation 1](#_Toc216954403)

[CURED Toolbox 2](#_Toc216954404)

[CURED Usage 2](#_Toc216954405)

[**CURED_Main.py** 2](#_Toc216954406)

[**CURED_FindREs.py** 3](#_Toc216954407)

[Wet Lab Protocol for NICU Cluster 1 4](#_Toc216954408)

[A. Primer Design 4](#_Toc216954409)

[B. Detailed Protocol 4](#_Toc216954410)

[C. Amplification Troubleshooting 5](#_Toc216954411)

[Methods of Figure 2 5](#_Toc216954412)

[Panel A 5](#_Toc216954413)

[Methods of Supplementary Figure 1 6](#_Toc216954414)

[Methods of Supplementary Figure 2 6](#_Toc216954415)

[Methods of Supplementary Figure 3 7](#_Toc216954416)

[References 7](#_Toc216954417)

[Supplementary Figure 1. 8](#_Toc216954418)

[Supplementary Figure 2. 8](#_Toc216954419)

[Supplementary Figure 3. 9](#_Toc216954420)

[Supplementary Figure 4. 10](#_Toc216954421)

[Supplementary Tables’ Legends 10](#_Toc216954422)

[Supplementary Table 1. 10](#_Toc216954423)

[Supplementary Table 2. 10](#_Toc216954424)

[Supplementary Table 3. 10](#_Toc216954425)

## Installation

**Cloning the GitHub repository:**

*git clone https://github.com/microbialARC/CURED
cd CURED
conda env create -f cured.yml
conda activate cured
chmod u+x bin/*
cp bin/* "$CONDA_PREFIX"/bin/*

**Installing with Bioconda:**

*conda install bioconda::cured*

**Installing with Docker:**
*docker pull theillere/cured:1.0.5
docker load -i cured-1.0.5.tar.gz (Only use this if you are loading from a local file)
docker run -it cured:1.0.5
CURED_Main.py -h*

## CURED Toolbox

The CURED pipeline consists of two scripts:

1. **CURED_Main.py**: The purpose of this script is to identify any sequences that are unique to the designated case group. The sensitivity and specificity thresholds can be defined by the user. The default is 100% sensitivity and 100% specificity.
2. **CURED_FindREs.py**: The purpose of this script is to identify any unique restriction enzyme sites in any of the unique k-mers. The specificity for finding these sites can be set by the user. By default, specificity is set to 100%, meaning that by finding the same enzyme site in just one control precludes the restriction site from being unique.

## CURED Usage

### **CURED_Main.py**

1. **DEPENDENCY:**
   1. Mlst (tested with version 2.23.0)
   2. Ncbi-datasets (tested with version 15.28.0)
   3. Unitig-caller (tested with version 1.3.0)
2. **INPUT:**
   1. Case/control file
   2. Genomes folder
3. **COMMAND LINE OPTIONS:**

Running CURED with a locally curated dataset:

*CURED_Main.py --case_control_file case_control_input.csv --genomes_folder /path/to/genomes/*

Running CURED by selecting a species of interest and a sequence type of interest:

*CURED_Main.py --species Staphylococcus aureus --sequence_type 300*

Running CURED using a list of accession numbers to be used as the case group:

*CURED_Main.py --species Pseudomonas putida --case_accession_list case_accessions.txt --database GenBank*

Running CURED using a list of accession numbers to be used as case and controls:

*CURED_Main.py --case_control_file case_control_accessions.txt --use-datasets*

Running CURED by providing a folder of local genomes to be used as cases and download a species to be used as controls:

*CURED_Main.py --case_genomes --genomes_folder genomes/ --species Anaplasma phagocytophilum --database GenBank*

Running CURED in simple mode, by providing a list of known k-mers to be queried against a set of genomes:

*CURED_Main.py --use_simple --kmer_list kmers_to_query.txt --case_control_file case_control.txt --genomes_folder genomes/*

1. **OUTPUT :**
   1. **UniqueKmers.txt:** a list of k-mers found within the set sensitivity and specificity thresholds. This can be used directly with CURED_FindREs.py
   2. **Unique_Kmers_Report.txt:** report of unique k-mers within the set sensitivity and specificity thresholds. Details the number of controls and/or cases that each k-mer is found in.

### **CURED_FindREs.py**

1. **DEPENDENCY:**
   1. samtools (tested with version 1.18)
   2. blast (tested with version 2.15.0)
   3. bwa (tested with version 0.7.17)
   4. biopython (tested with version 1.82)
2. **INPUT:**
   1. Case/control file
   2. Unique k-mers
   3. Genomes folder
3. **COMMAND LINE OPTIONS:**

Running CURED_FindREs.py with default parameters:

*CURED_FindREs.py --case_control_file case_control_input.csv UniqueKmers.txt /path/to/genomes/*

Running CURED_FindREs.py in full-support mode:

*CURED_FindREs.py --case_control_file case_control_input.csv --specificity 0 UniqueKmers.txt /path/to/genomes/*

Running CURED_FindREs.py in coordinate comparison mode:

*CURED_FindREs.py --compare_coordinates UniqueKmers.txt /path/to/genomes/*

Running CURED_FindREs.py in coordinate comparison mode and full-support mode:

*CURED_FindREs.py --compare_coordinates --specificity 0 UniqueKmers.txt /path/to/genomes/*

1. **OUTPUT**:
   1. **CURED_UniqueEnzymes.tsv:** Report of unique restriction sites in the k-mers.
   2. **CURED_FindREs_controls.txt:** If a restriction enzyme site that is found in the case genomes is identified in any of the control genomes, the names of the controls are reported with the corresponding k-mer and restriction enzyme.
   3. **CURED_FindREs_summary.txt**: Summary report detailing the k-mer, case genome used, number of control genomes used in the search and the number of control genomes excluded due to no alignment found, or low coverage.
   4. **CURED_UniqueEnzymes_PCR_Products.tsv:** If a restriction enzyme is identified as unique to the case, the PCR product for the corresponding k-mer is outputted.

The *in-silico* PCR target region is outputted by CURED for further downstream work. Output files are available online to download (1). The time required to run each of these commands can be found in **Supplementary Table 3**.

## Wet Lab Protocol for NICU Cluster 1

### Primer Design

We designed primers for each k-mer for Cluster 1 using the Primer3web 4.1.0 software (2), positioning primers a minimum of 100 bp upstream and downstream of the k-mer, with final distances determined by local sequence composition and primer quality metrics. The k-mers were then ranked based on primer melting temperature, GC content, the efficiency of the restriction enzyme associated with the k-mer (as inferred from recognition sequence length, with preference for longer cutters such as 6-base recognition sites), and the sizes of the pre- and post-digested products, using the SnapGene 8.0.3 software (3), to ensure clear separation on a 1.5% agarose gel.

### Detailed Protocol

BD BBL™ CultureSwab™ EZ collection and transport system are used to screen the nares of inpatients in the NICU at CHOP’s Main Hospital (~600 beds) and then plated to Blood Agar Plate and BBLTM CHROMagarTM MRSA II. All growth on CHROMagar suspected to be MRSA (mauve colonies) is confirmed to be *S. aureus* with the Staphaurex™ Latex Agglutination Test and then subculture onto blood agar and confirmed to be MRSA with ClearView® PBP2a, a Rapid Lateral Flow Assay. Growth of characteristic *S. aureus* colonies on blood agar without CHROMagar is indicative of MSSA. All suspected MSSA are confirmed to be *S. aureus* with the Staphaurex™ Latex Agglutination Test and confirmed to not produce PBP2A with the ClearView® PBP2a, a Rapid Lateral Flow Assay. For each confirmed *S. aureus,* we used pipet tips to gently touch the edge of 3 separate colonies and crushed each colony into 0.2uL microcentrifuge tubes. We used the New England Biolabs (NEB) Taq 5X Master Mix (Cat: M0285L) at 25uL final volume to amplify the selected k-mer region for cluster 1 from the primer design step on the Eppendorf nexus gradient mastercycler using the suggested thermocycling conditions, except for a modified 5-minute initial denaturation step to help break open the cell wall and release the DNA. Primer annealing temperature was calculated using NEB Tm calculator at 500nM final primer concentration (4).

As an alternative to direct colony-based PCR, we also tested a crude DNA extraction of colonies using the Direct from Colony Extraction protocol from Illumina. Briefly, using a 10 µl disposable inoculation loop, a half loopful of colonies from the bacterial blood culture plate were picked and resuspend in the Qiagen PowerBead tubes (Cat: 13116-50) containing 0.5mm glass beads and nuclease-free water. Bead beating step was then done on the Qiagen TissueLyser III (Cat: 9003240), followed by a magnetic bead cleanup (Mag-Bind Total Pure NGS, Omega Bio-tek (Cat: M1378-01) and Paragon Genomics 96 well CleanMag magnetic plate (Cat: 719002)) of the extracted DNA. We used 10uL of a 1:10 dilution of the extracted, cleaned DNA as the input volume for k-mer amplification. When extracted DNA is used as sample input, the initial 95°C denaturation can be switched to 30 seconds, instead of 5 minutes. Post-amplification, we performed restriction enzyme digestion on half of the amplified product without a prior buffer exchange or clean up step. We added 0.5uL of AvaII (Cat: R0153S) restriction enzyme and 2.5uL of the rCutSmart buffer to 12.5uL of the amplified product, followed by 1.5-hours digestion. The reaction was then heat-inactivated at 80°C for 20 minutes; all programed on the Eppendorf nexus gradient mastercycler. Volumes and times for the restriction enzyme digestion were optimized to generate products that would produce easily interpretable bands when visualized on a gel. The digested products were run on a 1.5% agarose gel at 100V for 30 minutes and the gel was imaged on an Azure Biosystems C600 gel imager at UV365 with auto-exposure. For larger sample volume, we automated our bench workflow by using the Eppendorf epMotion 5075 Liquid Handler to set up reaction plates for k-mer amplification and downstream restriction digestion. We used the QIAxcel Connect System (Cat: 9003110) to perform capillary electrophoresis of the digested samples and generated a report that identified samples as belonging to cluster 1 based on the expected sizes of the cut bands.

### Amplification Troubleshooting

Of the 45 samples tested with the CURED protocol, two failed to amplify. Sequencing confirmed these samples were *S. aureus*, but mutations in the primer binding sites prevented primer binding and subsequent amplification (**Supplementary Figure 3**). Consequently, cluster membership could not be determined for these isolates using this protocol.

## Methods of Figure 2

### Panel A

To benchmark CURED’s computational performance (runtime and memory usage), a custom Python script was used to randomly select 100, 1000, 10000, 20000, 40000 and 79754 genomes from the control group of the RdJ dataset to pair with the case group. For simplicity, the 12 control genomes that were identified as having the RdJ k-mer were excluded. Note, iteration_num was changed to randomly select 100, 1000, 10000, 20000, 40000 and 79754. The names of the different strains used in each dataset are listed in **Supplementary Table 1**. All benchmarking was performed on a Linux machine with access to 32 CPU cores and 140 GB of RAM. To ensure consistent performance comparisons across tools, analyses were restricted to a single CPU core.

*python3 randomly_subset_data.py batching_case_control_all.txt iteration_num controls_with_kmer.txt*

For each tool, the analysis was run on the selected dataset three times. KEC version 1.0 and KmerGO version 1.5 were used. The following commands were used to run the respective tool:

**KEC:** *kec-v1-linux-x64 exclude -n nontarget_79754/ -t target/ -k 20 -o all.iteration1*

**KmerGO:** *KmerGO_for_cmd -m 0 -k 20 -assn 1.0 -ci 1 -t traits.csv -i genomes/*

**CURED – 8 threads:** *CURED_Main.py --number_of_cases 400 --genomes_folder genomes/ --threads 8 --number_of_controls 1000 --case_control_file 79754_case_control.txt*

**CURED – 1 thread:** *CURED_Main.py --genomes genomes/ --case_control_file 20000_case_control.txt*

Memory and time were recorded in the **Supplementary Table 1**. These were used to create the plot in Panel B with a custom R script.

## Methods of Supplementary Figure 1

GCF_902703115.1 was randomly selected from the case-designated genomes and annotated using bakta (5). The 91 unique k-mers that were identified by CURED_Main.py were queried against GCF_902703115.1 using blastn and converted to BED format:

*blastn -query biomarkers.fasta -subject GCF_902703115.1.fna -percent_identity 100 -word_size 10 -evalue 1e-2 -outfmt 6 -out blast_results.tsv*

*awk '{print $2 "\t" $9-1 "\t" $10 "\t" $1 "\t" $12 "\t" ($9<$10?"+":"-")}' blast_results.tsv > blast_results.bed*

The U1 k-mer identified by Álvarez, Verónica Elizabeth, et al was queried against GCF_902703115.1 using blastn (6) with default parameters, and the results were converted to BED format.

*blastn -query U1.fa -subject GCF_902703115.1.fna -outfmt 6 -out U1_blast.tsv*

*awk '{print $2 "\t" $9-1 "\t" $10 "\t" $1 "\t" $12 "\t" ($9<$10?"+":"-")}' U1_blast.tsv > U1.bed*

The U1 k-mer was identified in the moaCB gene by Álvarez, Verónica Elizabeth, et al. The moaCB gene was identified in the GFF3 file and extracted using samtools (7). The extracted gene was queried against GCF_902703115.1 using blastn with default parameters and converted to BED format.

*samtools faidx GCF_902703115.1.fna contig_8: 19784-20707 > moaCB.fna*

*blastn -query moaCB.fa -subject GCF_902703115.1.fna -outfmt 6 -out moaCB_blast.tsv*

*awk '{print $2 "\t" $9-1 "\t" $10 "\t" $1 "\t" $12 "\t" ($9<$10?"+":"-")}' moaCB_blast.tsv > moaCB.bed*

GCF_902703115.1.fna and its corresponding index file were loaded into IGV. The three BED files were loaded as tracks in Integrated Genomics Viewer (8).

## Methods of Supplementary Figure 2

GCF_000003215.1 was randomly selected from the case-designated genomes. The 10 unique k-mers identified by CURED_Main.py were queried against GCF_000003215.1 using blastn (6) and converted to BED format.

*blastn -query biomarkers_blast_input.fa -subject GCF_000003215.1.fna -percent_identity 100 -word_size 10 -evalue 1e-2 -outfmt 6 -out blast_results.tsv*

*awk '{print $2 "\t" $9-1 "\t" $10 "\t" $1 "\t" $12 "\t" ($9<$10?"+":"-")} blast_results.tsv > biomarker_blast_results.bed*

Using tcdA and tcdB as a proxy for the pathogenicity locus, these genes were queried against GCF_000003215.1 using blastn (6) and converted to BED format.

*blastn -query toxin_genes.fa -subject GCF_000003215.1.fna -outfmt 6 -out blast_results.tsv*

*awk '{print $2 "\t" $9-1 "\t" $10 "\t" $1 "\t" $12 "\t" ($9<$10?"+":"-")} blast_results.tsv > toxin_genes.bed*

samtools (7) was used to index GCF_000003215.1.

*samtools faidx GCF_000003215.1.fna*

GCF_000003215.1.fna and its corresponding index file were loaded into IGV (8). The two BED files were loaded as tracks in IGV.

## Methods of Supplementary Figure 3

To investigate amplification failure in two NICU samples, we aligned corresponding genomic regions of the two samples to the CURED primer sequences using AliView (9).

## References

1. Theiller E, Moustafa A. Classification Using Restriction Enzyme Diagnostics (CURED): Additional Files [Data set]. Zenodo. 2025; <https://doi.org/10.5281/zenodo.15597023>.

2. Untergasser A, Cutcutache I, Koressaar T, Ye J, Faircloth BC, Remm M, et al. Primer3--new capabilities and interfaces. Nucleic Acids Res. 2012;40(15):e115.

3. SnapGene software: [www.snapgene.com](file:///Users/theillere/Documents/www.snapgene.com) [

4. Tm Calculator: <https://tmcalculator.neb.com/#!/main> [

5. Schwengers O, Jelonek L, Dieckmann MA, Beyvers S, Blom J, Goesmann A. Bakta: rapid and standardized annotation of bacterial genomes via alignment-free sequence identification. Microb Genom. 2021;7(11).

6. Altschul SF, Gish W, Miller W, Myers EW, Lipman DJ. Basic local alignment search tool. J Mol Biol. 1990;215(3):403-10.

7. Danecek P, Bonfield JK, Liddle J, Marshall J, Ohan V, Pollard MO, et al. Twelve years of SAMtools and BCFtools. Gigascience. 2021;10(2).

8. Robinson JT, Thorvaldsdottir H, Winckler W, Guttman M, Lander ES, Getz G, et al. Integrative genomics viewer. Nat Biotechnol. 2011;29(1):24-6.

9. Larsson A. AliView: a fast and lightweight alignment viewer and editor for large datasets. Bioinformatics. 2014;30(22):3276-8.

10. Alvarez VE, Quiroga MP, Centron D. Identification of a Specific Biomarker of Acinetobacter baumannii Global Clone 1 by Machine Learning and PCR Related to Metabolic Fitness of ESKAPE Pathogens. mSystems. 2023;8(3):e0073422.


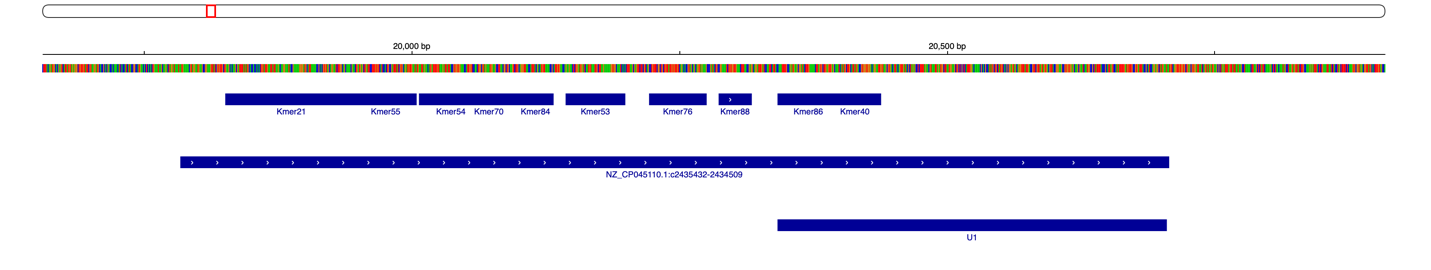


U1

*moaCB*

Unique k-mers identified by CURED

Supplementary Figure 1. Alignment image from Integrated Genomics Viewer (IGV) showing the k-mers identified by CURED (top track) that are overlapping with the U1 region from Álvarez, Verónica Elizabeth, et al. The U1 region was identified in the *moaCB* gene (8, 10).

*
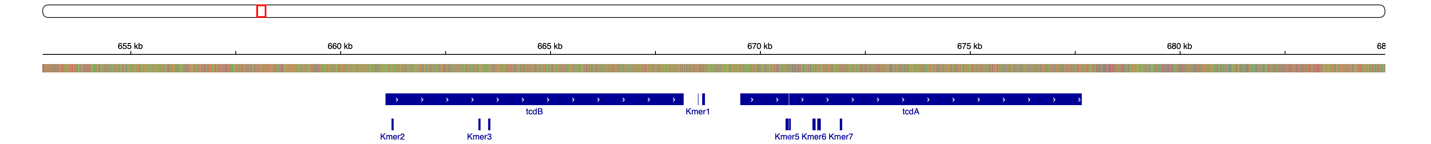
*

*tcdB* and *tcdA* genes

Unique k-mers identified by CURED

Supplementary Figure 2. Alignment image from IGV showing the unique k-mers identified by CURED in reference to GCF_000003215.1, a toxigenic *C*. *difficile* genome. The toxigenic genes, *tcdB* and *tcdA* are visualized as reference points (8).

Supplementary Figure 3. Example Fragment analysis of 13 CHOP NICU surveillance samples using capillary electrophoresis. Uncut bands indicate absence of the biomarker k-mer, while digested bands confirm the presence of Cluster 1-associated sequences.

Supplementary Figure 4. Alignment in AliView showing mutations in the primer binding sites for Sample 1 and Sample 2, which failed to amplify using the CURED bench protocol. The forward primer is indicated by the top box, and the reverse primer by the bottom box. Pink dots indicate positions where the sequence matches the corresponding nucleotide.

Supplementary Tables’ Legends

Supplementary Table 1. Runtime metrics (time (d-h:m:s) and memory in GBs) for CURED, KEC and KmerGO, with corresponding genome accession numbers used in benchmarking (xlsx).

Supplementary Table 2. K-mer results from running CURED with USA300 *Staphylococcus aureus* dataset, with corresponding genome accession numbers used in benchmarking (xlsx).

Supplementary Table 3. Genome accession numbers used in benchmarking for *Acinetobacter*, *C. difficile*, NICU datasets (xlsx).
